# Venetoclax with decitabine versus decitabine monotherapy in elderly acute myeloid leukemia: a propensity score-matched analysis

**DOI:** 10.1038/s41408-022-00770-x

**Published:** 2022-12-19

**Authors:** Daehun Kwag, Byung-Sik Cho, Su-Yeon Bang, Jong Hyuk Lee, Gi-June Min, Sung-Soo Park, Silvia Park, Jae-Ho Yoon, Sung-Eun Lee, Ki-Seong Eom, Yoo-Jin Kim, Seok Lee, Chang-Ki Min, Seok-Goo Cho, Jong Wook Lee, Hee-Je Kim

**Affiliations:** 1 Department of Hematology, Catholic Hematology Hospital, Seoul St. Mary’s Hospital, College of Medicine, The Catholic University of Korea, Seoul, Republic of Korea; 2 Leukemia Research Institute, College of Medicine, The Catholic University of Korea, Seoul, Republic of Korea

**Keywords:** Acute myeloid leukaemia, Acute myeloid leukaemia

## Abstract

Venetoclax (VEN) combined with azacitidine (AZA) or decitabine (DEC) has been approved for older adults with acute myeloid leukemia (AML) unfit for intensive chemotherapy based on the pivotal VIALE-A trial. However, this trial only compared AZA + VEN with AZA monotherapy. Therefore, we compared the outcomes of consecutive older adults (65 years or older) with newly diagnosed AML who received DEC (*n* = 230) or DEC + VEN (*n* = 74) after propensity score matching to construct a one-to-one matched cohort by the nearest neighbor algorithm. The median overall survival was longer in the DEC + VEN group than in the DEC group (13.4 months vs. 8.3 months, *p* = 0.01). The median event-free survivals were 8.6 and 5.8 months in the DEC + VEN and DEC groups, respectively (*p* = 0.02). The response rate (complete response, complete response with incomplete hematologic recovery, and morphologic leukemia-free state) was significantly higher in the DEC + VEN group than in the DEC group (70.3% vs. 24.3%, *p* < 0.01). The 30-day (2.7% vs. 9.5%, *p* = 0.17) and 60-day (9.5% vs. 18.9%, *p* = 0.16) mortality rates did not differ between the two groups, nor did the median hospitalization and transfusion rates (hospitalization: 23 days vs. 21 days, *p* = 0.20; red blood cells: 3.2 units/month vs. 3.5 units/month, *p* = 0.73; platelets: 2.7 units/month vs. 2.3 units/months, *p* = 0.48). Of those who received DEC + VEN and became leukemia-free, 29% underwent allogeneic stem cell transplantation and had excellent survival outcomes (one-year survival: 79.4%; one-year non-relapse mortality: 13.3%). This study is the first to provide real-world evidence that DEC + VEN has superior outcomes to DEC monotherapy.

## Introduction

Older adults with acute myeloid leukemia (AML) have inferior survival outcomes compared to younger patients since they often have diseases with higher risk factors, a decreased performance status, and acquired comorbidities [1]. Older adults with AML unfit for intensive chemotherapy usually tolerate hypomethylating agents (HMA), such as azacitidine (AZA) and decitabine (DEC), resulting in lower treatment-related mortality (TRM) rates. However, HMAs only provide modest response and survival rate benefits compared to low-dose cytarabine; the complete response (CR) plus CR with incomplete hematologic recovery (CRi) rates are less than 30%, and the median overall survival (OS) is less than one year [2, 3].

B-cell lymphoma-2 (BCL-2) is an anti-apoptotic protein that allows cancer cells to evade apoptosis by sequestering pro-apoptotic proteins [4]. Moreover, it is highly expressed in leukemic blasts and stem and progenitor cells [5]. Venetoclax (VEN) is a potent and selective small-molecule BCL-2 inhibitor [6], and VEN monotherapy had a modest efficacy for relapsed and refractory AML [7]. However, a preclinical AML model identified synergistic activity between VEN and HMAs [8], and a phase 1 study in older adults with newly diagnosed AML unfit for intensive chemotherapy found that VEN combined with azacitidine (AZA) or decitabine (DEC) had high durable response rates [9, 10]. Based on these results, the combination therapy of HMA (AZA or DEC) and VEN obtained accelerated approval by the Food and Drug Administration (FDA) in 2018, and the FDA granted regular approval of HMA + VEN through the results of pivotal VIALE-A, which reported a CR plus CRi of 66.4% and a median OS of 14.7 months in the AZA + VEN group compared to 28.3% and 9.6 months, respectively, in the AZA monotherapy group [11]. However, the FDA approved DEC + VEN without a phase 3 study, and none have compared DEC + VEN with DEC monotherapy. Moreover, the DEC + VEN data were limited owing to a small number of cases that were analyzed together with the AZA + VEN cases [12, 13]. Therefore, this study compared DEC + VEN with DEC monotherapy in a propensity-matched cohort including older adults with newly diagnosed AML to clarify effects of adding VEN to DEC therapy.

## Methods

### Patients and treatment regimen

This single-center, retrospective study enrolled newly diagnosed patients with AML aged 65 years or older who received DEC + VEN or DEC monotherapy as front-line treatment from February 2013 to December 2021. None of these patients received DEC or DEC + VEN treatment in a clinical trial or any other targeted agents. DEC monotherapy consisted of administering 20 mg/m^2^ intravenous DEC daily for five days. The response was evaluated through a bone marrow (BM) study after cycle 2 unless disease progression was firmly not suspected in cycle 1. If the patient remained stable, the subsequent BM evaluation was performed after cycle 4, then every four cycles after that. The DEC + VEN group received the same daily dose of DEC for five days combined with 28 days of VEN (400 mg daily) per cycle. The cycle 1 ramp-up, disease response evaluation, VEN dose reduction in concomitant with azoles, and the resting period between cycles were the same as in the VIALE-A trial [11, 14, 15]. We induced cytoreduction of white blood cell (WBC) counts <20 × 10^9^/L by hydroxyurea, low-dose intravenous cytarabine, or leukapheresis before DEC or DEC + VEN treatment. Uric acid reducing agents (allopurinol, febuxostat, or rasburicase) and intravenous fluid hydration were administered for the prevention of tumor lysis syndrome. We used fluconazole 400 mg/day during the neutropenic period in all patients as antifungal prophylaxis without antibacterial prophylaxis [14, 15]. DEC and DEC + VEN treatments were maintained until disease progression, a severe adverse event, a hematopoietic stem cell transplantation (HSCT), or the patient requested discontinuation. If the patient achieved a CR, became fit for HSCT, or wanted to proceed to the HSCT, then HSCT was performed. The Institutional Review Board of Seoul St. Mary’s Hospital approved this study, which followed the principles of the Declaration of Helsinki.

### Propensity score matching

VEN has been available in our institution for patients with AML aged 65 years or older since March 2020. Before the introduction of DEC + VEN, DEC monotherapy was the standard treatment for older adults with AML unfit for intensive chemotherapy. Therefore, we constructed a propensity score-matched cohort to minimize bias between the DEC + VEN and DEC groups. We calculated the propensity score using a logistic regression model that included age, performance status (determined by the Eastern Cooperative Oncology Group [ECOG] Status Scale), karyotype (grouped by the Medical Research Council criteria; Supplementary Table 1) [16], etiology (*de novo* or secondary AML), WBC count, platelet count, blast cells in peripheral blood (%), and the serum albumin and creatinine levels. These variables are known prognostic factors associated with early death and OS [17], especially in older adults with AML [18]. We extracted patients from the DEC monotherapy group (n = 230) by one-to-one propensity score matching with the DEC + VEN group (*n* = 74) using the nearest-neighbor algorithm. After matching, we confirmed that the matched cohort was well balanced through the standardized mean difference of the calculated variables [19]. Supplementary Fig. 1 summarizes the propensity score matching process.

### Measurable residual disease (MRD) assessment

We assessed MRD status before HSCT using real-time quantitative polymerase chain reaction (PCR) assays (Bioseum, Seoul, Korea) for *NPM1, RUNX1::RUNX1T1*, and *CBFB::MYH11* in the *NPM1*-mutated or core binding factor AML patients, and the transcripts level of *Wilms tumor gene 1* in the other patients, as previously described [20]. Mutated genes were calculated with standard materials and normalized with respect to the number of *ABL1* transcripts and expressed as copy numbers per 1 × 10^5^ copies of *ABL1*. Assays were performed in replicate with appropriate controls. Limit of detection was evaluated at the time of test setting and determined as 1 × 10^−5^.

### Statistics

Baseline characteristics or values are presented as medians with interquartile ranges (IQRs) or counts with percentages. We used the European Leukemia Net (ELN) 2010 classification to categorize the patient’s genetic risk group since a full mutation profile was lacking in the DEC group [21]. The Wilcoxon rank-sum, and chi-square tests were used to compare categorical and continuous variables, respectively. We compared OS and event-free survival (EFS), defined as the duration from the start of the DEC + VEN or DEC until disease progression, relapse from a leukemia-free state (CR, CRi, or morphologic leukemia-free state), death, or censorship, between the DEC + VEN and matched-cohort DEC group. In the survival analysis, we generated a stratified Cox model using the propensity score strata as a stratifying variable [22]. Survival outcomes were compared within each subgroup categorized by the criteria proposed by Wheatley et al. (poor or not) [18] and the TRM score grouping suggested by Walter et al. (high or not) [17], which are validated prognostic and TRM risk evaluating tools in older adults with AML, respectively. The treatment response was evaluated based on the ELN 2017 recommendations [23]. The initial response, best response, and the time and cycles to reach the best response among patients who reached a leukemia-free state were compared between the two groups. We also compared the duration of response (DOR; from response [CR/CRi/MLFS] to death or relapse) [24]. Furthermore, in the treatment period which was determined from the treatment initiation to the end of treatment owing to disease progression, relapse, death, or a regimen change due to a lack of response or intolerance, total hospitalization days (excluding hospitalization for drug administration), transfusion requirement (red blood cells [RBC] and platelets), and transfusion independence (defined as no requirement for RBC or platelet transfusion for at least for two months during treatment period) were compared between the two groups. Exploratory subgroup analyses were performed in patients who proceeded to allogeneic HSCT after achieving a leukemia-free state by DEC + VEN treatment, and for the entire cohort to identify differences in the response or survival between the two treatment groups based on the baseline characteristics. All analyses were performed using R software (version 4.0.3, R Foundation for Statistical Computing, Vienna, Austria). *P*-values < 0.05 were considered statistically significant.

## Results

### Baseline characteristics

Table 1 presents the baseline characteristics for each group, which were well-balanced. The overall median age was 71 years (IQR: 68–76); 28.4% of patients in the DEC + VEN group and 32.4% in the DEC group were 75 years or older (*p* = 0.72). The number of patients with decreased performance (ECOG score of 2 or higher) (17.6% vs. 25.7%, *p* = 0.32) or secondary AML (both groups, 28.4%, *p* = 1.0) did not differ between the groups. Furthermore, the WBC and platelet counts, peripheral blood chemistry findings, and BM blast rates at diagnosis were similar between the groups. The cytogenetic and genomic risk distributions also did not differ; 23% of patients in both groups were classified with adverse risk. Both groups had a similar proportion of patients with poor risk based on the Wheatley index (DEC + VEN: 58.1%, DEC: 60.8%, *p* = 0.87) and high-risk based on the TRM score (DEC + VEN: 56.8%, DEC: 62.2%, *p* = 0.62) [17, 25]. Conversely, the number of patients who underwent HSCT significantly differed between the groups (DEC + VEN: 19 patients [25.7%], DEC: 4 patients [5.4%], *p* < 0.01). Of them, two DEC + VEN patients switched to other treatments before the HSCT due to intolerance, and 2 others did not reach remission before the HSCT; one had a partial response, and the other reached a stable disease state. All patients were in remission before the HSCT in the DEC group.

**Table 1:** Baseline characteristics of patients.

Variables	DEC (N: 74)	DEC + VEN (N: 74)	*p*
Age, years (Median, IQR)	72 (70–76)	71 (68–75)	0.10
Age ≥ 75 years (*N*, %)	24 (32.4)	21 (28.4)	0.72
Male (*N*, %)	37 (50.0)	32 (43.2)	0.51
ECOG ≥ 2 (*N*, %)	19 (25.7)	13 (17.6)	0.32
Secondary AML (*N*, %)	21 (28.4)	21 (28.4)	1
BM blast, % (median, IQR)	58.5 (38.0–81.5)	51 (30.8–77.2)	0.50
WBC > 25 × 10^9^/L (*N*, %)	20 (27.0)	18 (24.3)	0.85
Platelets < 30 × 10^9^/L (*N*, %)	12 (16.2)	20 (27.0)	0.16
Albumin < 3.5 g/dL (*N*, %)	11 (14.9)	8 (10.8)	0.62
Creatinine > 1.2 mg/dL (*N*, %)	8 (10.8)	5 (6.8)	0.56
Prothrombin time, INR > 1.2 (*N*, %)	24 (32.4)	25 (33.8)	1
Cytogenetics (MRC risk) (*N*, %)			0.96
Favorable	6 (8.1)	7 (9.5)	
t(8;21)(q22;q22)	4 (5.4)	3 (4.1)	
inv(16)(p13q22)	2 (2.7)	4 (5.4)	
Intermediate	51 (68.9)	50 (67.6)	
Adverse	17 (23.0)	17 (23.0)	
*FLT3-ITD* mutated (*N*, %)	9 (12.2)	6 (8.1)	0.59
*NPM1* mutated (*N*, %)	17 (23.0)	12 (16.2)	0.41
*CEBPA* mutated (*N*, %)	7 (9.5)	5 (6.8)	0.76
ELN 2010 risks (*N*, %)			0.54
Favorable	12 (16.2)	12 (16.2)	
Intermediate-l	20 (27.0)	27 (36.5)	
Intermediate-ll	25 (33.8)	18 (24.3)	
Adverse	17 (23.0)	17 (23.0)	
Poor Wheatley risk group * (*N*, %)	45 (60.8)	43 (58.1)	0.87
High TRM score group ** (*N*, %)	46 (62.2)	42 (56.8)	0.62
Proceed to HSCT (*N*, %)	4 (5.4)	19 (25.7)	<0.01

*AML* acute myeloid leukemia, *BM* bone marrow, *DEC* decitabine, *ECOG* Eastern Cooperative Oncology Group, *ELN* European leukemia net, *HSCT* hematopoietic stem cell transplantation, *INR* international normalized ratio, *MRC* Medical Research Council, *TRM* treatment-related mortality, *VEN* venetoclax, *WBC* white blood cell.

*Risk stratification proposed by Wheatley et al. [18].

**TRM score suggested by Walter et al. [17, 25].

### Treatment responses and early mortality

Table 2 presents the treatment response and early mortality results. Initially, 52.7% and 12.2% of patients in the DEC + VEN and DEC groups achieved a leukemia-free state (*p* < 0.01). Regarding the best response, significantly more patients in the DEC + VEN group obtained a leukemia-free state than in the DEC group (70.3% vs. 24.3%, *p* < 0.01). In the DEC + VEN patients, three patients were MLFS status at the best response, and their initial responses were MLFS, partial response, and stable disease, respectively. One of the two patients whose initial response was MLFS improved to CR after the second cycle (Supplementary Fig. 2). The duration until a leukemia-free state (median months and cycles) was significantly shorter in the DEC + VEN group than in the DEC group (1.3 months vs. 3.5 months, *p* < 0.01; 1 cycle vs. 4 cycles, *p* < 0.01; Supplementary Fig. 2), and of those achieving leukemia-free, DOR was longer in the DEC + VEN group than in the DEC group but statistically insignificant (13.8 months vs. 6.8 months, *p* = 0.10).

**Table 2:** Treatment response and early death.

	DEC (N: 74)	DEC + VEN (N: 74)	*p*
Initial response (*N*, %)			
Leukemia-free state	9 (12.2)	39 (66.2)	<0.01
CR/CRi	8 (10.8)	37 (50.0)	
CR	5 (6.8)	15 (20.3)	
CRi	3 (4.1)	22 (29.7)	
MLFS	1 (1.4)	2 (2.7)	
PR	1 (1.4)	9 (12.2)	
SD	32 (43.2)	19 (25.7)	
PD	8 (10.8)	1 (1.4)	
Death	24 (32.4)	6 (8.1)	
Best response (*N*, %)			
Leukemia-free state	18 (24.3)	52 (70.3)	<0.01
CR/CRi	17 (23.0)	49 (66.2)	
CR	9 (12.2)	26 (35.1)	
CRi	8 (10.8)	23 (31.1)	
MLFS	1 (1.4)	3 (4.1)	
PR	1 (1.4)	4 (5.4)	
SD	23 (31.1)	11 (14.9)	
PD	8 (10.8)	1 (1.4)	
Death	24 (32.4)	6 (8.1)	
Early death (*N*, %)			
30-day mortality	7 (9.5)	2 (2.7)	0.17
TRMS low (DEC: 28, DEC + VEN: 32)	5 (17.9)	0	0.02
TRMS high (DEC: 46, DEC + VEN: 42)	2 (4.3)	2 (4.8)	1
60-day mortality	14 (18.9)	7 (9.5)	0.16
TRMS low (DEC: 28, DEC + VEN: 32)	8 (28.6)	0	<0.01
TRMS high (DEC: 46, DEC + VEN: 42)	6 (13.0)	7 (16.7)	0.77
Duration of response, months (95% CI)*	6.8 (3.9-NA)	13.8 (5.8-NA)	0.10

*CR* complete remission, *CRi* complete remission with incomplete hematologic recovery, *DEC* decitabine, *MLFS* morphologic leukemia-free state, *NA* not available, *PD* progression of disease, *PR* partial response, *SD* stable disease, *TRMS* treatment-related mortality score suggested by Walter et al. [17, 25], *VEN* venetoclax.

*Duration of response was evaluated in the patients who achieved CR/CRi/MLFS.

Overall, the 30-day and 60-day mortality rates did not differ between the groups (30-day mortality: 9.5% vs. 2.7%, *p* = 0.17; 60-day mortality: 18.9% vs. 9.5%, *p* = 0.16). There were two deaths within 30 days due to pneumonia with sepsis and tumor lysis syndrome, respectively, in the DEC + VEN group, and 5 additional deaths (one in CRi, and the others in no hematologic response after the first cycle) by infection occurred until day 60. In the DEC group, mortality was caused by infection, hemorrhage, and pulmonary thromboembolism (Supplementary Table 2). In the low TRM risk group, the early mortality rate was lower in the DEC + VEN group than in the DEC group (30-day mortality: 0% vs. 17.9%, *p* = 0.02; 60-day mortality: 0% vs. 28.6% *p* < 0.01). However, the early mortality rate did not differ between the groups for high TRM risk patients (30-day mortality: 4.8% vs. 4.3%, *p* = 1.0; 60-day mortality: 16.7% vs. 13.0%, *p* = 0.77).

### Survival outcomes

**Fig. 1:**
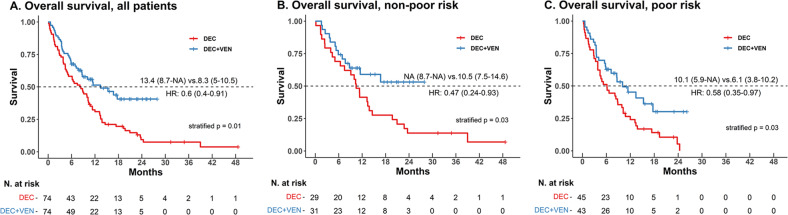
Overall survival, stratified by Wheatley risk groups. **A** Overall survival of all patients. **B** Overall survival of non-poor Wheatley risk patients. **C** Overall survival of poor Wheatley risk patients. DEC decitabine, HR hazard ratio, NA not available since the median was not reached, VEN venetoclax.

The median follow-up of the patients in the DEC + VEN group and DEC group was 8.3 months and 8.5 months, respectively, and the median follow-up of surviving patients in each group (DEC + VEN, *n* = 37; DEC, *n* = 7) was 11.5 months and 22.3 months. OS was significantly longer in the DEC + VEN group than in the DEC group (median OS: 13.4 months vs. 8.3 months, hazard ratio [HR]: 0.60, 95% confidence interval [CI]: 0.40–0.91, *p* = 0.01, Fig. 1A). We also compared the OS censored at the time of the HSCT since significantly different proportions of patients underwent HSCT per group; OS was better in the DEC + VEN group than in the DEC group (median OS: 15.3 months vs. 8.2 months, HR: 0.62, 95% CI: 0.40–0.97, *p* = 0.03, Supplementary Fig. 3A).

**Fig. 2:**
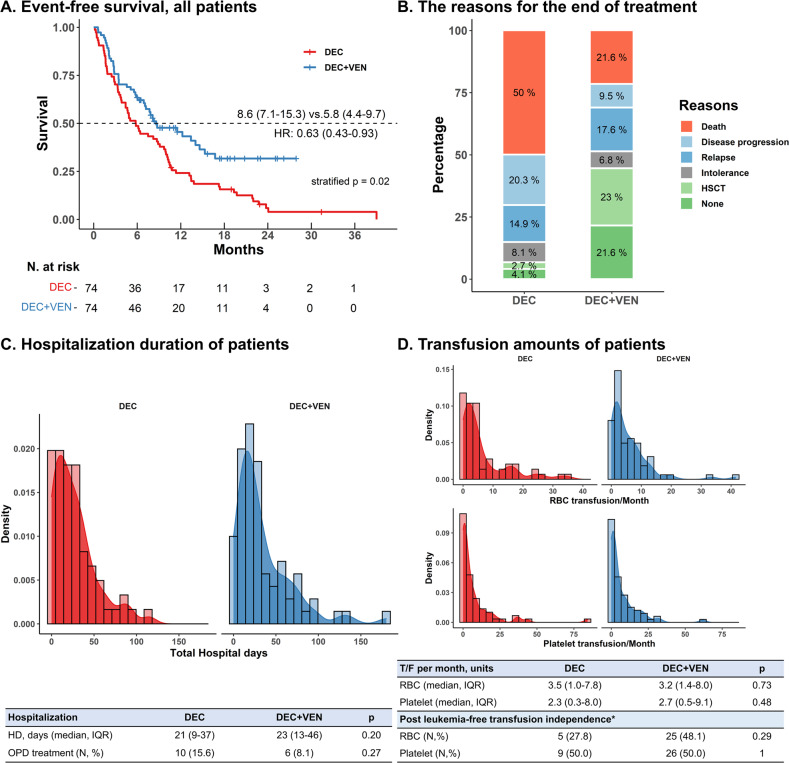
Various treatment outcomes of patients. **A** Event-free survival of whole matched patients. **B** The reasons for the end of treatment in both treatment groups. **C** Hospitalization duration of patients. **D** RBC/platelet transfusion requirement of patients. *In patients who obtained a leukemia-free state. DEC decitabine, EFS event-free survival, F/U follow-up, HD hospitalization days, HR hazard ratio, HSCT hematopoietic stem cell transplantation, IQR interquartile range, OPD outpatient department, RBC red blood cell, T/F transfusion, VEN venetoclax.

OS was significantly longer in the DEC + VEN group than in the DEC group regardless of the Wheatly index risk (median OS: non-poor risk: NA [Not achieved] vs. 10.5 months, HR: 0.47, 95% CI: 0.24–0.93, *p* = 0.03, Fig. 1B; poor risk: 10.1 months vs. 6.1 months, HR 0.58: 95% CI: 0.35–0.97, *p* = 0.03, Fig. 1C). Furthermore, OS censored at the time of the HSCT had a similar trend, with better outcomes in the DEC + VEN group (Supplementary Fig. 3B, C). Similarly, OS was better in the DEC + VEN group than in the DEC group for patients with low TRM risk (median OS: NA vs. 8.7 months, HR: 0.30, 95% CI: 0.14–0.63, *p* < 0.01). However, the groups did not differ for patients with high TRM risk (median OS: 8.7 months vs. 8.3 months, HR: 0.70, 95% CI: 0.43–1.14, *p* = 0.22, Supplementary Fig. 4). Finally, the EFS was better in the DEC + VEN group than in the DEC group (median 8.6 months vs. 5.8 months, *p* = 0.02, Fig. 2A).

We also evaluated the reasons for the end of treatment (Fig. 2B); the most common reason in the DEC + VEN group was HSCT (23.0%), followed by non-relapse/progression-related mortality (NRM; 21.6%). The disease progression and relapse rates in the DEC + VEN group were 9.5% and 17.6%, respectively. In the DEC group, NRM was most common (50.0%), followed by disease progression (20.3%) and relapse (14.9%). Five patients (6.8%) in the DEC + VEN group and six (8.1%) in the DEC group changed regimens due to intolerance. Among them, one DEC + VEN patient requested to discontinue the treatment due to cost but not drug-induced adverse events.

### Hospital stay duration and transfusions

During the treatment period, the hospital stay duration (excluding admission for drug administration) was similar between the groups. (DEC + VEN vs. DEC: median stay: 23 days vs. 21 days, *p* = 0.20), as was the proportion of outpatient-treated patients without hospitalization (DEC + VEN vs. DEC: 8.1% vs. 15.6%, *p* = 0.27, Fig. 2C). The transfusion requirement per month during treatment period was also comparable between the groups (DEC + VEN vs. DEC: median RBC transfusion per month: 3.2 units vs. 3.5 units, *p* = 0.73; median platelet transfusion per month: 2.7 units vs. 2.3 units, *p* = 0.48). Among those who achieved a leukemia-free state, transfusion independence did not differ between the groups (DEC + VEN vs. DEC: RBC: 48.1% vs. 27.8%, *p* = 0.29; platelets, 50.0% vs. 50.0%, *p* = 1.0; Fig. 2D).

### Survival outcomes of DEC + VEN patients who underwent allogeneic HSCT

Of 52 leukemia-free patients in the DEC + VEN group, 15 (28.8%) underwent allogeneic HSCT. The time from DEC + VEN treatment to HSCT was 5.1 (range, 3.6–11.1) months. The median age was 67 (range, 65–72) years, and the ELN 2017 risk distribution was 73.3% and 26.7% in the intermediate and adverse risk groups, respectively. The MRD negative remission was 60.0%. Approximately half of the donors were haploidentical (53.3%), followed by matched unrelated (33.3%), matched sibling (6.7%), and mismatched unrelated (6.7%). Most patients (91.9%) received reduced-intensity conditioning. The one-year OS rate after HSCT was 79.4%, and no patient died within 100 days after HSCT (Supplementary Fig. 5). The one-year relapse and NRM rates were 20.0% and 13.3%, respectively.

### Whole-cohort subgroup analyses

We analyzed the best treatment response (achieving leukemia-free state or not) and the OS of all included patients (*n* = 304) based on the disease subgroup to determine whether the VEN + DEC treatment differentially affected patients with various disease subtypes (Supplementary Figs. 6 and 7). Regarding the best response, the VEN benefit was identifiable regardless of age, leukocytosis, percentage of BM blasts, disease type, and karyotype. However, the benefits were not observed for patients with poorer ECOG scores (Supplementary Fig. 6). Similar trends were observed for the survival outcomes, except for a poor ECOG score and non-*de novo* AML. Notably, markedly superior survival of patients in the DEC + VEN group compared to the DEC group was observed for those in the poor karyotype and adverse ELN 2010 groups (Supplementary Fig. 7).

The response patterns among groups with specific gene mutations also demonstrated significantly better response rates in the DEC + VEN group than in the DEC group for patients with *FLT3-ITD*, *NPM1*, *CEBPA*, and *DNMT3A* mutations (Supplementary Fig. 6). However, these better responses did not translate into significant survival improvements in our cohort (Supplementary Fig. 7). In the DEC + VEN group, patients with *FLT3-ITD* (83.3%), *NPM1* (91.7%), *CEBPA* (80.0%), *DNMT3A* (80.0%), *DDX41* (80.0%), and *IDH2* (100%) mutations had excellent responses, but those with *NRAS* (33.3%), *KRAS* (0%) *TP53* (50.0%), *KIT* (50.0%), *RUNX1* (28.6%), and *ASXL1* (50.0%) mutations had suboptimal responses (Supplementary Fig. 8).

Finally, we analyzed the prognostic role of the entire cohort’s two indexes (the Wheatley index and TRM score) per treatment group (Supplementary Fig. 9). In the DEC group, the Wheatley index poor risk group had significantly worse OS than the non-poor risk group (median 6.8 months vs. 12.0 months, *p* < 0.01). However, the OS did not differ based on the TRM risk (low vs. high: median 9.9 months vs. 8.6 months, *p* = 0.96). In the DEC + VEN group, OS did not differ between the Wheatley index poor risk and non-poor risk groups (NA vs. 10.1 months, *p* = 0.12), but the OS was significantly worse in the high TRM risk group than in the low TRM risk group (low vs. high: NA vs. 8.7 months, *p* = 0.02).

## Discussion

This study generated a propensity-matched cohort of older adults with newly diagnosed AML based on relevant factors to minimize the effect of confounders and bias inherent in retrospective comparisons [26–28]. We found that DEC + VEN significantly improved the response rates and survival outcomes compared to DEC monotherapy. Both groups were balanced based on the Wheatley index, which is a prognostic model validated for older patients treated with low-intensity regimens and intensive chemotherapy [18], and the TRM score, which was developed in a cohort of primarily intensively treated AML patients [17]. Adding VEN to DEC therapy did not affect the hospital stay duration or transfusion requirements. Furthermore, 29% of patients who reached a leukemia-free state with DEC + VEN treatment underwent allogeneic HSCT and had excellent survival outcomes. Currently, no data compare DEC + VEN treatment to DEC monotherapy. Therefore, this is the first study to demonstrate the effects of adding VEN to DEC therapy. Our data provide strong evidence to support the validity of the FDA’s approval of DEC + VEN. These results are important since the FDA approved DEC + VEN without the supporting data; the VIALE-A trial only compared AZA + VEN to AZA monotherapy.

Other landmark studies have also combined VEN with HMAs for older adults with newly diagnosed AML [11, 29]. In our study, DEC + VEN (*n* = 74) had similar response rates and survival outcomes to the results of previous studies, such as DEC + VEN (*n* = 31) and AZA + VEN (*n* = 84) in a phase 1b HMA + VEN study (400 mg dose of VEN) and AZA + VEN (*n* = 286) in the VIALE-A study (Supplementary Table 3). The DEC + VEN group in our study was relatively younger, had better ECOG performance scores, and fewer patients had a poor karyotype; all our DEC + VEN patients of poor karyotype by MRC criteria corresponded to adverse karyotype by ELN criteria. Despite those favorable characteristics, our patients in the DEC + VEN group had relatively shorter median OS compared to AZA + VEN in the phase 1b and VIALE-A trial (13.4 months vs. 16.4 months vs. 14.7 months, respectively). These could be explained by a shorter follow-up duration of our patients (median follow-up duration; current study vs. phase 1b vs. VIALE-A; 8.3 months vs. 29 months vs. 20.5 months) and the differences between real-world data and clinical trials. In addition, the distribution of other prognostic variables should be considered for comparison. From this perspective, approximately half of our study were classified as high-risk groups based on validated prognostic models, such as the Wheatley index (58.1%) and the TRM score (56.8%), which was incomparable due to the lack of this information in the phase 1b and VIALE-A trial [11, 29].

Exploratory analyses in the VIALE-A study suggest significantly superior outcomes in patients with *IDH*-mutated AML treated with AZA + VEN compared to AZA monotherapy, which aligns with our data. Despite the small number of patients, we found high response rates for DEC + VEN patients with *IDH*-mutated AML. Furthermore, DEC + VEN resulted in excellent responses for patients with *FLT3-ITD*, *NPM1*, *CEBPA*, *DNMT3A*, and *DDX41* mutations. However, patients with *NRAS*, *KRAS*, *TP53*, *KIT*, *RUNX1*, and *ASXL1* mutations had suboptimal responses. In the survival analyses in our study, patients with a poor karyotype had better outcomes with DEC + VEN than with DEC monotherapy. However, in the VIALE-A study, patients with an intermediate karyotype had better survival outcomes with AZA + VEN than with AZA monotherapy. We could not perform multivariate analysis and conclude the sensitivity of DEC and AZA combined with VEN against specific mutations and karyotypes due to a limited number of patients treated by DEC + VEN, warranting further large-scale studies. Nonetheless, DEC + VEN had favorable outcomes compared to DEC monotherapy in this study, similar to the outcomes of AZA + VEN, suggesting equal reliability between the two. Additionally, the VIALE-A study only included a small number of Asian individuals (AZA + VEN: *n* = 48; AZA: *n* = 26) and did not demonstrate superior survival after AZA + VEN treatment in this subgroup [11]. Our study clarifies the survival benefit of HMA + VEN treatments in elderly Asian patients with AML.

Interestingly, the DEC + VEN group had better OS than the DEC group, regardless of the Wheatley index risk assessment. However, the survival benefit of DEC + VEN was not observed in the high TRM risk group. Moreover, the Wheatley index score was not associated with OS in the DEC + VEN group but was significantly associated with the DEC group. Conversely, the TRM score was significantly associated with OS in the DEC + VEN group but not in the DEC group. Regarding early mortality, the 30-day mortality rate in DEC + VEN group (2.7%) was comparable to that of HMA + VEN in other clinical studies (DEC + VEN: phase1b 6.5%; AZA + VEN: phase 1b 2.4% & VIALE-A 7.4%) [11, 29]. Our study’s 60-day mortality rate of the DEC + VEN group was 9.5%, lower than that of DEC monotherapy (18.9%) without statistical significance. We could not compare the 60-day mortality with previous studies due to lacking HMA + VEN related data. Our data showed that deaths until day 60 in the DEC + VEN group occurred only in patients with high TRMS, whereas there was no early death in patients with low TRMS. This implies that the benefits of adding VEN to DEC in patients with high TRM risk are weakened by the significant risk of early death, highlighting the necessity of novel prognostic models according to the treatment type and intensity for older adults with newly diagnosed AML. Our results suggest that the TRM score may predict early death for patients receiving DEC + VEN treatment, similar to intensive chemotherapy, but this should be validated in a large cohort study.

A recent report indicated that newly diagnosed patients with AML treated with ten days of DEC with VEN (DEC10 + VEN) had better outcomes than those receiving intensive chemotherapy, particularly those at high risk of TRM [25, 30]. Our ability to directly compare their DEC10 + VEN and our DEC + VEN results is limited owing to differences in the clinical factors per cohort (Supplementary Table 4), but we found that slightly more patients reached a leukemia-free state after DEC10 + VEN treatment (81%) than after DEC + VEN treatment (70.3%) [25]. However, the OS was similar between the DEC + VEN and DEC10 + VEN groups (13.4 months vs. 12.4 months), despite a higher proportion of patients at high risk of TRM in our cohort (57% vs. 28%) [25]. Based on the TRM risk score, the 60-day mortality rate, CR/CRi, and OS did not differ between DEC10 + VEN and DEC + VEN groups, except for patients with a high TRM risk; they had a higher response rate with DEC10 + VEN (Supplementary Table 4). These results suggest that a ten-day DEC treatment combined with VEN may enhance response rate but not considerably improve the survival outcomes compared to a five-day DEC treatment. Given the age, ECOG performance, and TRM risk score differences between the two cohorts, randomized trials comparing DEC10 + VEN with DEC5 + VEN for older adults with newly diagnosed AML are warranted. In addition, DEC10 + VEN had better results than intensive chemotherapy in this patient population [25]. Therefore, trials comparing five days of DEC + VEN treatment with intensive chemotherapy are also necessary.

In total, 15 patients (29%) in the DEC + VEN group who became fit for allogeneic HSCT after achieving a leukemia-free state showed favorable outcomes, such as a one-year OS rate of 79.4%, a one-year relapse rate of 20.0%, and one-year NRM rate of 13.3%. Moreover, the HSCT results of patients receiving DEC + VEN were better than those who underwent allogeneic HSCT after intensive chemotherapy [31, 32], aligning with the results from other recent reports (Supplementary Table 5) [33, 34]. High rates of MRD negative remission before HSCT (60% in our cohort, 71.4% in Salhotra et al., and 59% in Kennedy et al.) by HMA + VEN treatment were sufficient to proceed to HSCT with reduced-intensity conditioning in older adults with AML [33, 34], suggesting that a VEN-based regimen may be a valuable bridging therapy for allogeneic HSCT in these elderly populations.

The current study has inherent limitations due to its retrospective design. We used propensity score matching to minimize the bias, but unexpected confounders could have influenced these results. The relatively short follow-up period is another potential limitation. However, the superior survival outcomes of the DEC + VEN group are unlikely to change because only a small number of patents were censored before the median survival point. In addition, our cohort is characterized by the high proportion of patients at high risk based on the Wheatley index and TRM scores, which are well-known prognostic models validated in older adults with AML. Finally, we could not conclude the outcome differences based on specific genomic abnormalities since a complete mutation profile was lacking in a subset of the DEC monotherapy group. Nonetheless, given the lack of prospective comparative data, the current study is the first to provide real-world evidence that DEC + VEN has superior outcomes to DEC monotherapy.

## Conclusion

DEC + VEN therapy had a better and faster treatment response than DEC monotherapy in older adults with newly diagnosed AML, resulting in superior OS and EFS without affecting the hospital stay or blood transfusion rates. Furthermore, DEC + VEN is a feasible bridge to an allogeneic HSCT in this population, providing strong evidence for choosing not only AZA but also DEC when combined with VEN. Finally, our data highlight the necessity of novel prognostic models for patients treated with VEN-based regimens and clinical trials comparing DEC + VEN and DEC10 + VEN or intensive chemotherapy treatments.

## Supplementary information

Supplemental material

## Data Availability

Please contact the corresponding author (cbscho@catholic.ac.kr) to access the original data.
