# Supplementary material for: Venetoclax with decitabine versus decitabine monotherapy in elderly acute myeloid leukemia: a propensity score-matched analysis
Source: Blood Cancer J. 2022 Dec 19;12(12):169. doi: 10.1038/s41408-022-00770-x (PMC9760636; doi:10.1038/s41408-022-00770-x)
Supplement: Supplementary file 1 — Supplemental material [file 41408_2022_770_MOESM1_ESM.docx]

# Supplementary Table 1. Karyotype classification of MRC and ELN criteria

|  | **MRC criteria*** | **ELN 2010**** | **ELN 2022***** |
| --- | --- | --- | --- |
| **Favorable** | t(15;17)(q22;q21)  t(8;21)(q22;q22)  inv(16)(p13q22)/t(16;16)(p13;q22) | t(8;21)(q22;q22.1)  inv(16)(p13.1q22) or t(16;16)(p13.1;q22) | t(8;21)(q22;q22.1)  inv(16)(p13.1q22) or t(16;16)(p13.1;q22) |
| **Intermediate**† | Entities not classified as favorable or adverse | t(9;11)(p21.3;q23.3)  Cytogenetic abnormalities not classified as favorable or adverse | t(9;11)(p21.3;q23.3)  Cytogenetic abnormalities not classified as favorable or adverse |
| **Adverse** | abn(3q) [excluding t(3;5)(q21~25;q31~35)]  inv(3)(q21q26)/t(3;3)(q21;q26)  add(5q), del(5q),5  -7, add(7q)/del(7q),  t(6;11)(q27;q23),  t(10;11)(p11~13;q23)  t(11q23) [excluding t(9;11)(p21~22;q23) and t(11;19)(q23;p13)]  t(9;22)(q34;q11)  17/abn(17p)  Complex (≥ 4 unrelated abnormalities) | inv(3)(q21.3q26.2) or t(3;3)(q21.3;q26.2)  t(6;9)(p23;q34.1)  t(v;11)(v;q23)  -5 or del(5q); -7; abnl(17p)  Complex karyotype‡ | t(6;9)(p23;q34.1)  t(v;11q23.3)  t(9;22)(q34.1;q11.2)  t(8;16)(p11.2;p13.3)  inv(3)(q21.3q26.2) or t(3;3)(q21.3;q26.2)  t(3q26.2;v)  -5 or del(5q); -7;-17/abn(17p)  Complex karyotype§  Monosomal karyotype¶ |

ELN: European Leukemia Net; MRC: Medical Research Council. *Grimwade et al. [15] **Döhner et al. [19] ***Döhner et al. Blood 2022;140:1345–1377. †In ELN 2010, Intermediate-1 and intermediate-2 karyotypes are combined. ‡Three or more chromosome abnormalities in the absence of one of the WHO designated recurring translocations or inversions, that is, t(15;17), t(8;21), inv(16) or t(16;16), t(9;11), t(v;11)(v;q23), t(6;9), inv(3) or t(3;3). §≥3 unrelated chromosome abnormalities in the absence of other class-defining recurring genetic abnormalities; excludes hyperdiploid karyotypes with three or more trisomies (or polysomies) without structural abnormalities. ¶Presence of two or more distinct monosomies (excluding loss of X or Y), or one single autosomal monosomy in combination with at least one structural chromosome abnormality (excluding core-binding factor AML).

# Supplementary Table 2. Causes of early mortality in DEC and DEC+VEN patients

| Causes of death* | Death in 30 days | | Death in 60 days | |
| --- | --- | --- | --- | --- |
| Number (N, %) | **DEC (N: 7)** | **DEC+VEN (N: 2)** | **DEC (N: 14)** | **DEC+VEN (N: 7)** |
| Sepsis | 2 (28.6) | 1 (50.0) | 6 (42.9) | 5 (71.4) |
| Pneumonia | 2 (28.6) | 1 (50.0) | 6 (42.9) | 4 (57.1) |
| Neutropenic enterocolitis |  |  | 1 (7.1) | 1 (14.3) |
| Fungal sinusitis | 1 (14.3) |  | 1 (7.1) |  |
| SSTI | 1 (14.3) |  | 4 (28.6) | 1 (14.3) |
| Brain hemorrhage | 2 (28.6) |  | 3 (21.4) |  |
| Pulmonary hemorrhage | 1 (14.3) |  | 1 (7.1) |  |
| Pulmonary thromboembolism | 1 (14.3) |  | 1 (7.1) |  |
| Tumor lysis syndrome |  | 1 (50.0) |  | 1 (14.3) |

DEC: Decitabine; SSTI: Skin and soft tissue infection; VEN: Venetoclax. *A single patient could be affected by multiple causes.

# Supplementary Table 3. Comparison of studies for DEC+VEN (400mg) with AZA+VEN (400mg)

|  | DEC+VEN | | AZA+VEN | |
| --- | --- | --- | --- | --- |
| Study | **Current study (2022)** | **Phase 1b* (2021)** | **Phase 1b* (2021)** | **VIALE-A** (2020)** |
| N. of patients | 74 | 31 | 84 | 286 |
| Age ≥ 75 years | 28.4% | 26% | 50% | 61% |
| ECOG ≥ 2 | 17.6% | 13% | 31% | 45% |
| Secondary AML | 28.4% | 29% | 25% | 25% |
| Poor karyotype | 23% | 48% | 39% | 36% |
| Median overall survival, months (95% CI) | 13.4 (8.7-NA) | 16.2 (9.1-27.8) | 16.4 (11.3-24.5) | 14.7 (11.9-18.7) |
| CR/CRi | 66.2% | 74% | 71% | 66.4% |
| CR/CRi after cycle 1 | 50.0% | 32% | 46% | 43.4% |
| MLFS | 4.1% | Not mentioned | Not mentioned | Not mentioned |
| Median duration of response, months (95% CI)^†^ | 13.8 (5.8-NA) | 15.0 (7.2-30.0) | 21.9 (15.1-30.2) | 17.5 (15.3-NA) |
| Median months to response | 1.3 | 1.9 | 1.2 | 1.3 |
| 30-day mortality | 2.7% | 6.5% | 2.4% | 7.4% |
| Transfusion independence |  |  |  |  |
| RBC | 48.1% | 61% | 64% | 59.8% |
| Platelet | 50.0% | 87% | 70% | 68.5% |

AZA: Azacitidine; CR: Complete remission; CRi: Complete remission with incomplete hematologic recovery; DEC: Decitabine; ECOG: Eastern Cooperative Oncology Group; MLFS: Morphologic leukemia-free state; RBC: Red blood cell; VEN: Venetoclax. *Pollyea et al. [29] **DiNardo et al. [11] †Duration of response in the current study was evaluated in the patients who achieved CR/CRi/MLFS, and defined as the time from response to relapse/progression or death [24].

# Supplementary Table 4. Comparison of DEC+VEN in the current study with a study for DEC10 (10 days of DEC) +VEN

|  | DEC+VEN | DEC10+VEN |
| --- | --- | --- |
| Study | Current study (2022) | Mahiti et al.* (2021) |
| N. of patients | 74 | 85 |
| Age ≥ 70 years | 55.4% | 74% |
| Age ≥ 80 years | 5.4% | 18% |
| Male | 43.2% | 53% |
| ECOG ≥ 2 | 17.6% | 35% |
| De novo AML | 71.6% | 65% |
| Poor karyotype | 23% | 47% |
| *FLT3* mutation | 8.1% (ITD) | 16% (ITD/TKD) |
| Proceed with HSCT | 25.7% | 14% |
| Median OS, months | 13.4 | 12.4 |
| CR/CRi | 66.2% | 81% |
| MLFS | 4.1% | 0 |
| High-risk TRMS† (N, %) | **42 (56.8)** | **24 (28.2)** |
| CR/CRi | 50.0% | 71% |
| MLFS | 7.1% | 8.3% |
| 30-day mortality | 4.8% | 0 |
| 60-day mortality | 16.7% | 17% |
| Median OS, months | 8.7 | 9.1 |
| Low-risk TRMS (N, %) | **32 (43.2)** | **61 (71.8)** |
| CR/CRi | 87.5% | 85% |
| MLFS | 0 | 4.9% |
| 30-day mortality | 0 | 2% |
| 60-day mortality | 0 | 3% |
| Median OS, months | NR | 15.2 |

CR: Complete remission; CRi: Complete remission with incomplete hematologic recovery; DEC: Decitabine; ECOG: Eastern Cooperative Oncology Group; HSCT: Hematopoietic stem cell transplantation; MLFS: Morphologic leukemia-free state; NR: Not reached; OS: Overall survival; TRM: Treatment-related mortality; VEN: Venetoclax. *Mahiti et al. [25] †TRM score suggested by Walter et al. [17]

# Supplementary Table 5. Comparison of studies for outcomes with HSCT at remission after frontline HMA+VEN

| Study | Current study (2022) | Salhorta et al.* (2022) | Kennedy et al.** (2022) |
| --- | --- | --- | --- |
| N. of patients | 15 | 21 | 46 |
| HMA type |  |  |  |
| AZA | 0 |  | 46% |
| DEC | 100% |  | 54% |
| Median age at HSCT, years | 67 |  | 70 |
| Male | 60% |  | 63% |
| De novo AML | 80% | 45% | 61% |
| Poor performance | 20% |  | 35% |
| ELN 2017 risk |  |  |  |
| Favorable | 0 |  | 17% |
| Intermediate | 73.3% |  | 28% |
| Adverse | 26.7% |  | 54% |
| *FLT3-ITD* mutation | 20% |  | 13% |
| *TP53* mutation | 13.3% |  | 17% |
| Response at HSCT |  |  |  |
| CR/CRi | 100% | 100% | 93% |
| MLFS | 0 | 0 | 7% |
| MRD† negativity | 60.0% | 71.4% | 59% |
| Myeloablative conditioning | 9.1% | 0 | 11% |
| PBSC graft | 100% | 100% | 96% |
| Donor type |  |  |  |
| MSD | 6.7% | 0 | 22% |
| MUD | 33.3% | 100% | 50% |
| MMUD | 6.7% | 0 | 0 |
| HID | 53.3% | 0 | 26% |
| Cord blood | 0 | 0 | 2% |
| Median survival | NR | NR | NR |
| 1-year OS | 79.4% | 85% | 73%* |
| 1-year relapse | 20.0% |  | 37.5%* |
| 1-year NRM | 13.3% | 12% | 17.0%* |

AZA: Azacitidine; CR: Complete remission; CRi: Complete remission with incomplete hematologic recovery; DEC: Decitabine; ELN: European Leukemia Net; HID: Haplo‑identical donor; HMA: Hypomethylating agent; HSCT: Hematopoietic stem cell transplantation; MLFS: Morphologic leukemia-free state; MMUD: Mismatched unrelated donor; MRD: Measurable residual disease; MSD: Matched sibling donor; MUD: Matched unrelated donor; NRM: Non-relapse mortality; OS: Overall survival; PBSC: Peripheral blood stem cell; VEN: Venetoclax. *Salhorta et al. [34] **Kennedy et al. [33] †MRD of the current study was evaluated by real-time quantitative PCR in *RUNX1-RUNX1T1*, *CBFB-MYH11*, and *NPM1* mutated patients. In the other patients, the transcripts level of *Wilms tumor gene 1* was used [20]. Salhorta et al. and Kennedy et al. assessed MRD via multiparametric flow cytometry [33, 34].

#
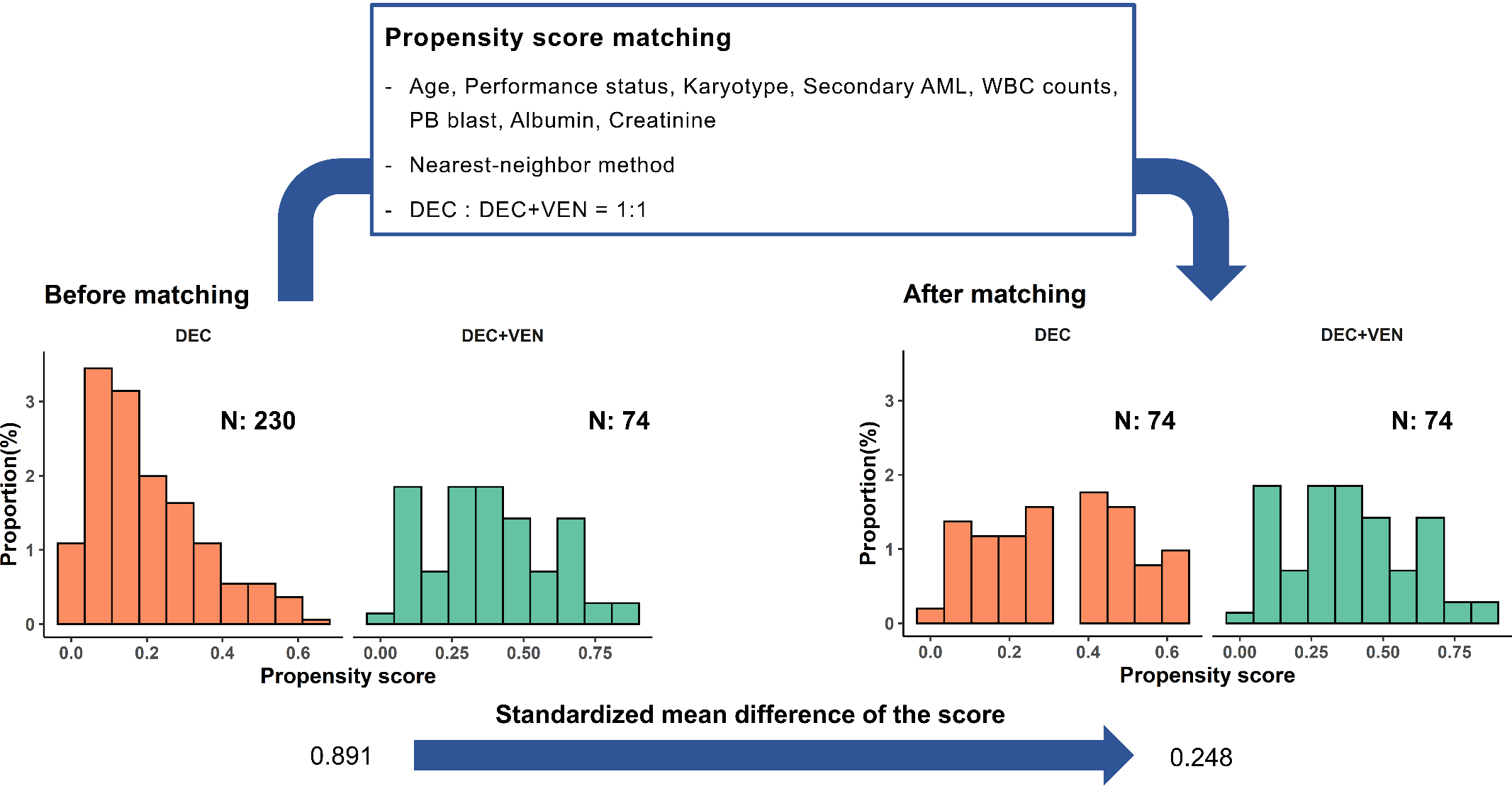
Supplementary Figure 1. Propensity score matching process. AML: Acute myeloid leukemia; DEC: Decitabine; PB: Peripheral blood; VEN: Venetoclax; WBC: White blood cell.

# Supplementary Figure 2. Treatment response of patients. Each patient’s initial response and best response are connected by a line. *Among patients who reached a leukemia-free state (CR, CRi, or MLFS). CR: Complete remission; CRi: Complete remission with incomplete hematologic recovery; DEC: Decitabine; IQR: Interquartile range; MLFS: Morphologic leukemia-free state; PD: Progression of disease; PR: Partial response; SD: Stable disease; VEN: Venetoclax


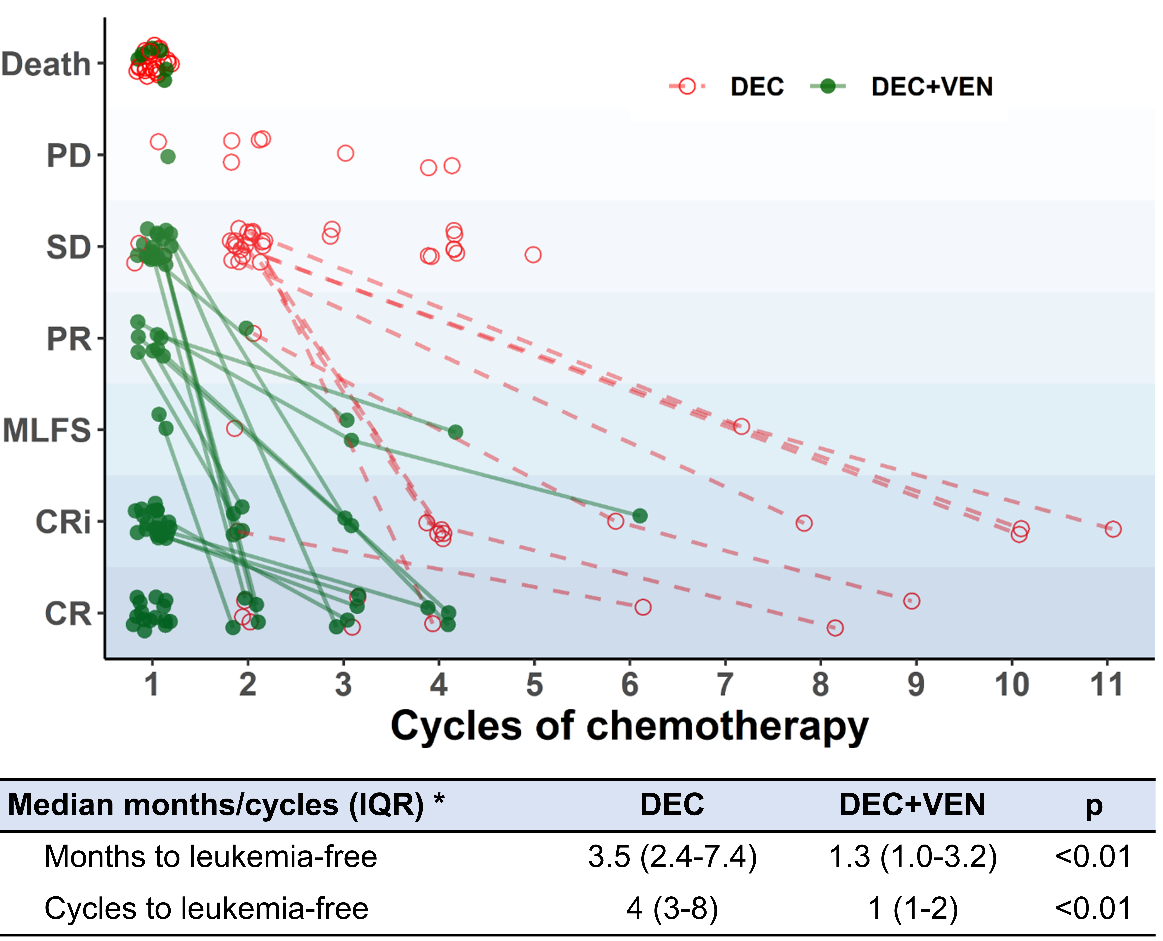


# Supplementary Figure 3. Survival censing at HSCT date, stratified by Wheatley risk groups. DEC: Decitabine; HR: Hazard ratio; HSCT: Hematopoietic stem cell transplantation; NA: Not available since the median was not reached; VEN: Venetoclax.


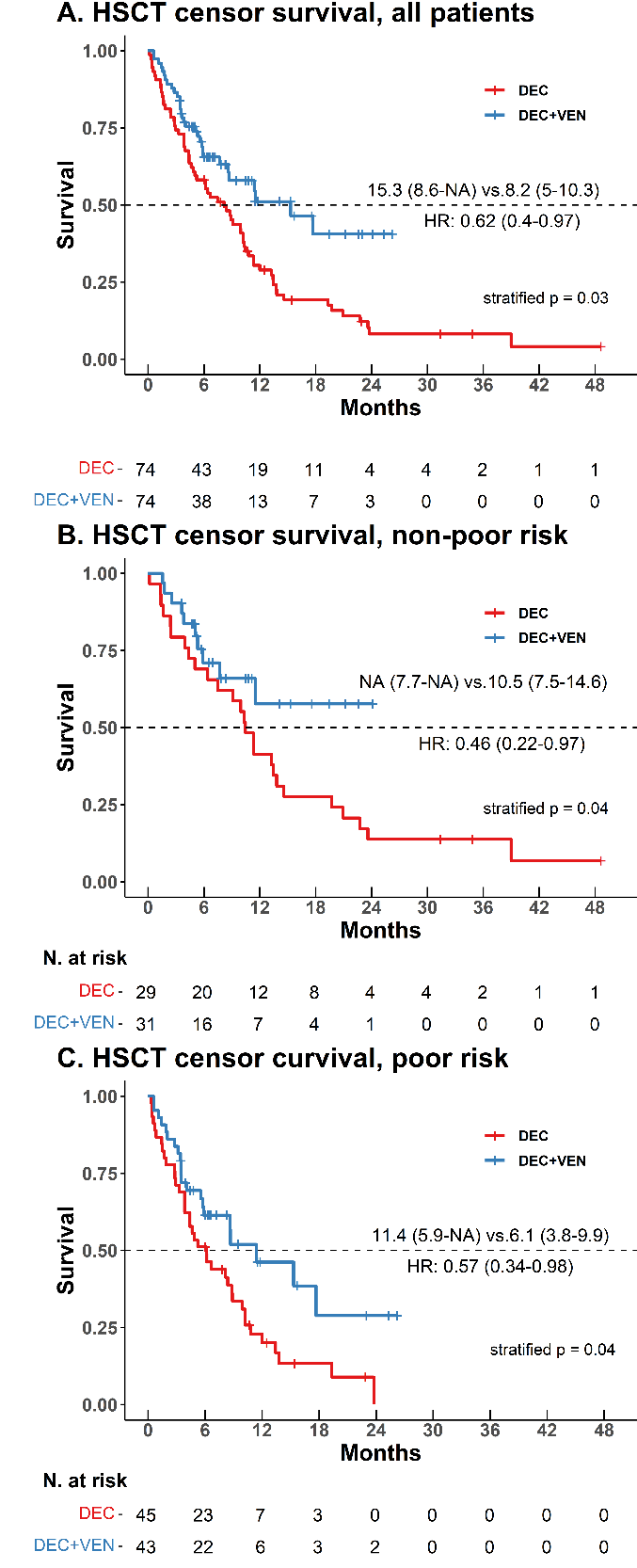


# Supplementary Figure 4. Overall Survival of patients according to TRM risk. DEC: Decitabine; HR; Hazard ratio; NA: not available since median survival was not reached; TRM: Treatment-related mortality; VEN: Venetoclax.


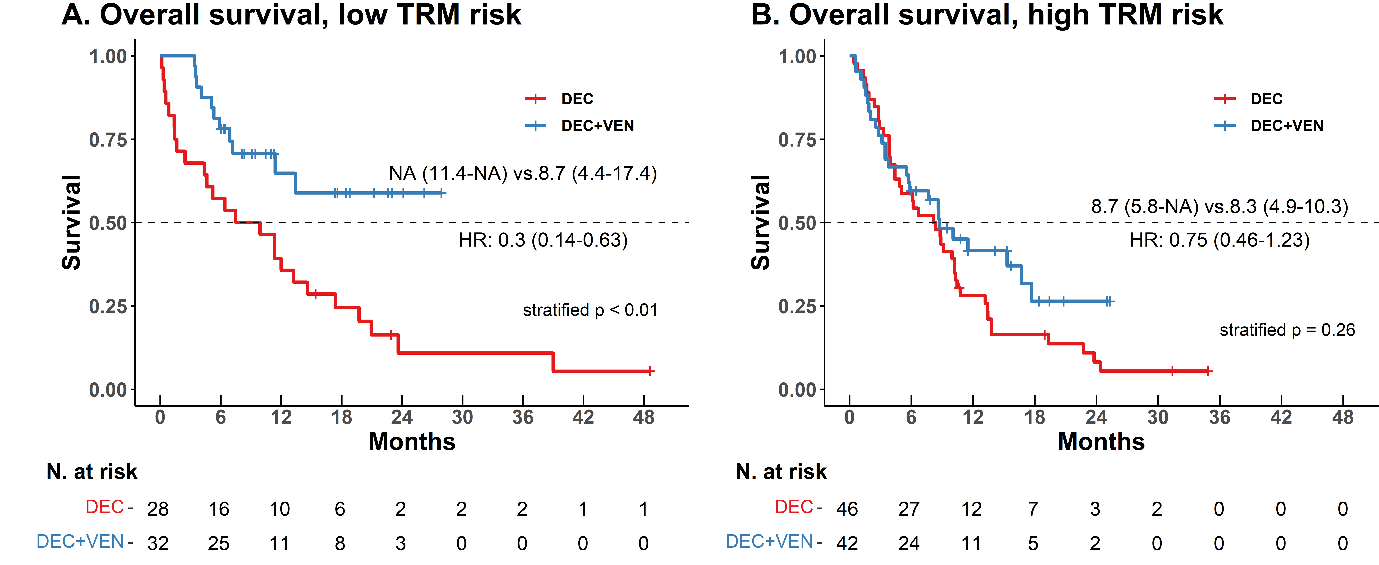


# Supplementary Figure 5. Survival after HSCT in DEC+VEN patients. DEC: Decitabine; HID: Haploidentical donor; HSCT: Hematopoietic stem cell transplantation; MMUD: Mismatched unrelated donor; MSD: Matched sibling donor; MUD: Matched unrelated donor; VEN: Venetoclax


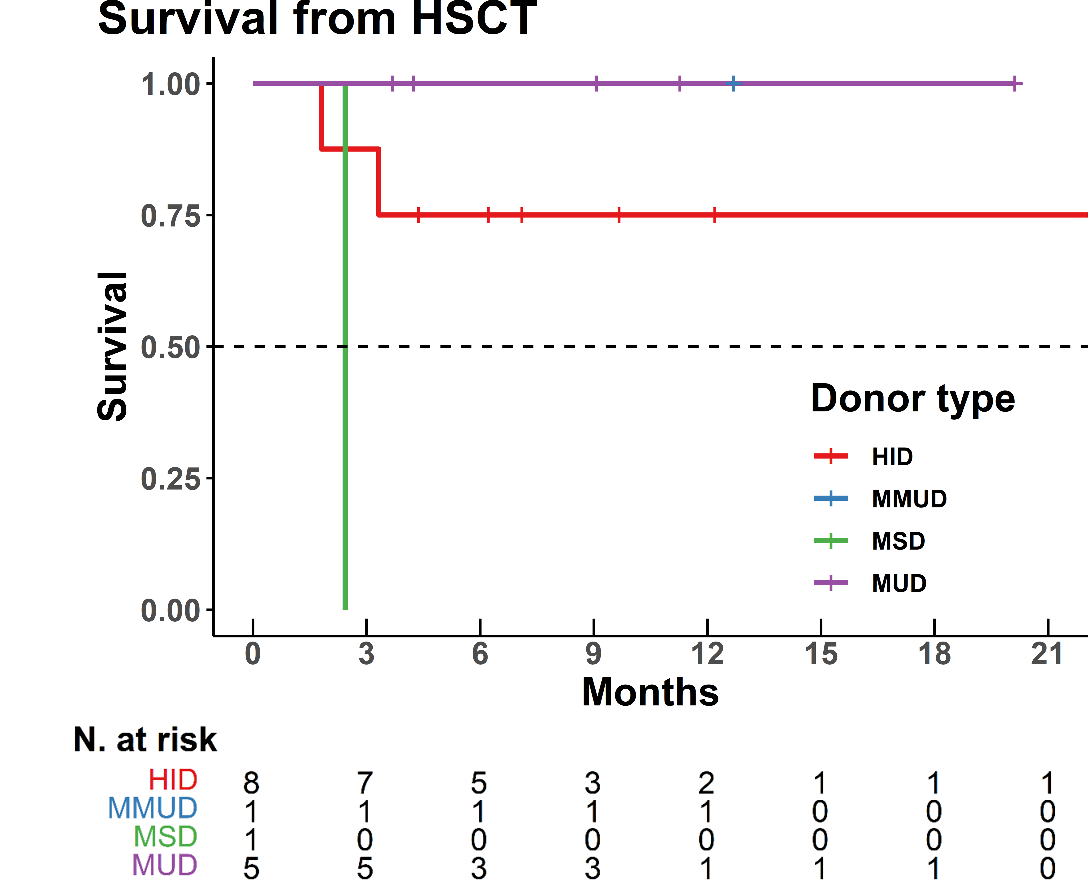

#
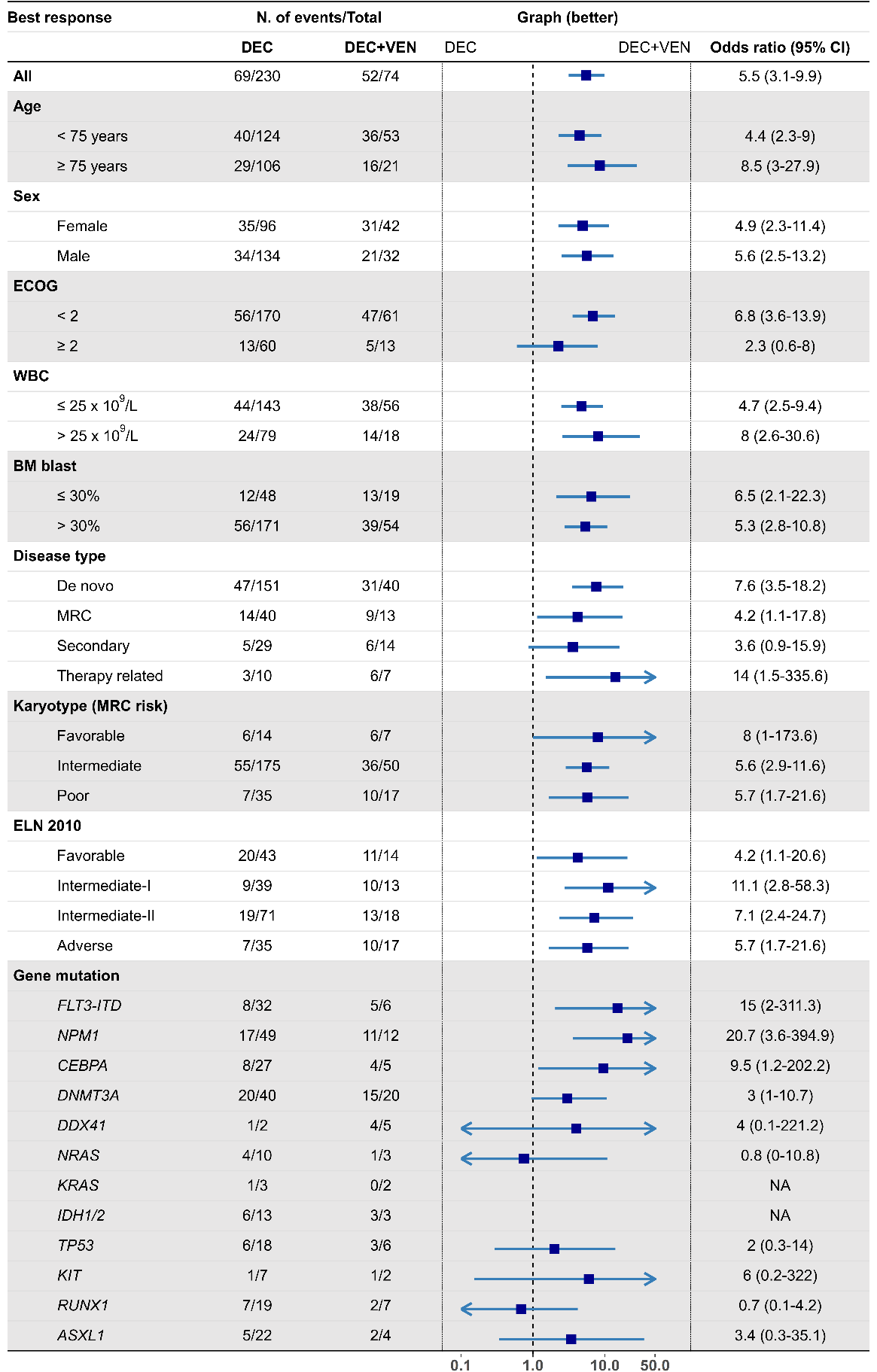
Supplementary Figure 6. Subgroup analysis of best treatment response in the whole cohort. Event means achieving a leukemia-free state. BM: Bone marrow; DEC: Decitabine; ECOG: Eastern Cooperative Oncology Group; ELN: European Leukemia Net; MRC (in disease type): Myelodysplasia related change; MRC (in karyotype): Medical Research Council; VEN: Venetoclax; WBC: White blood cell

#
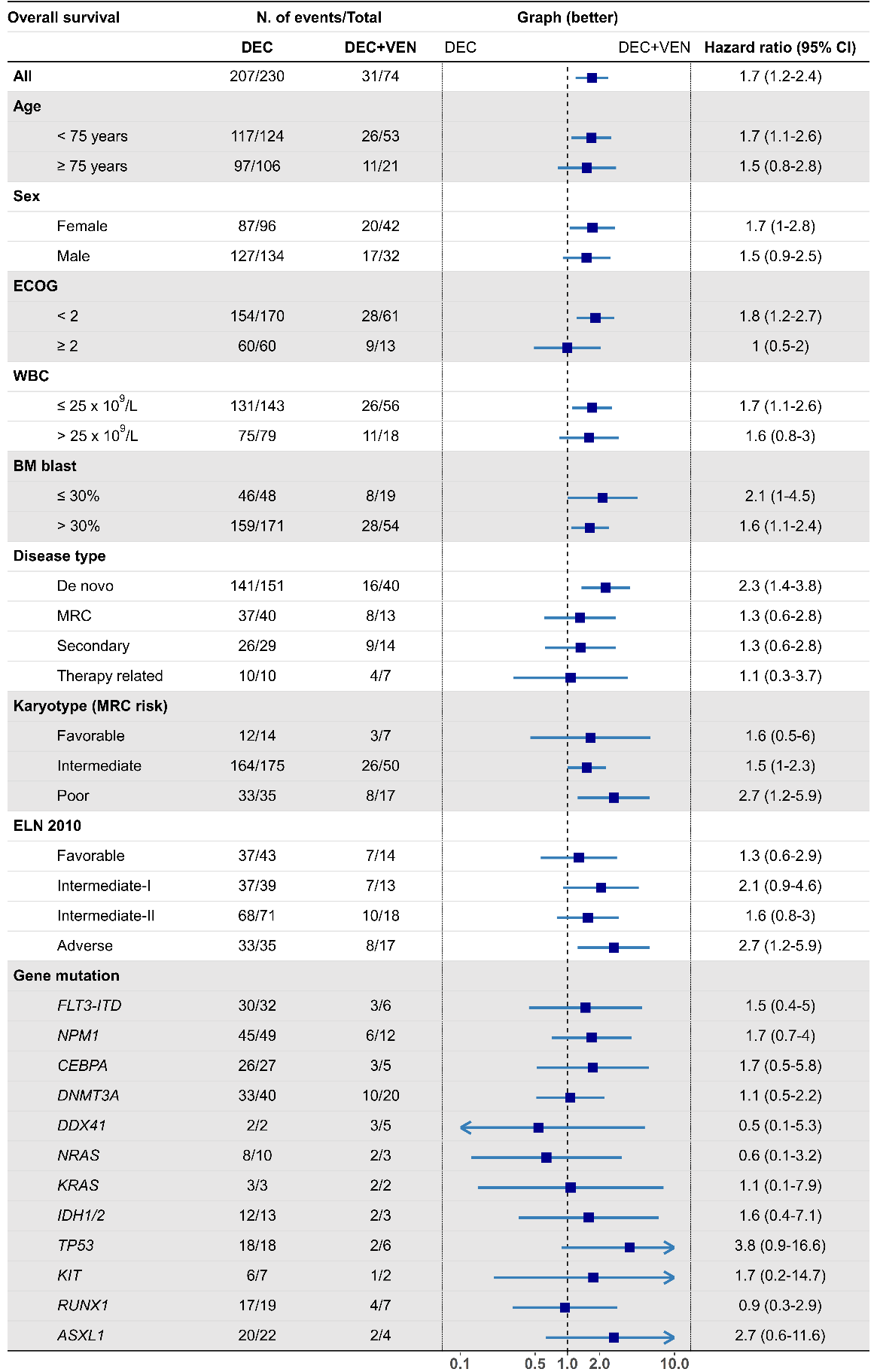
Supplementary Figure 7. Subgroup analysis of overall survival in the whole cohort. Event means death. BM: Bone marrow; DEC: Decitabine; ECOG: Eastern Cooperative Oncology Group; ELN: European Leukemia Net; HSCT: Hematopoietic stem cell transplantation; MRC (in disease type): Myelodysplasia related change; MRC (in karyotype): Medical Research Council; VEN: Venetoclax; WBC: White blood cell

#
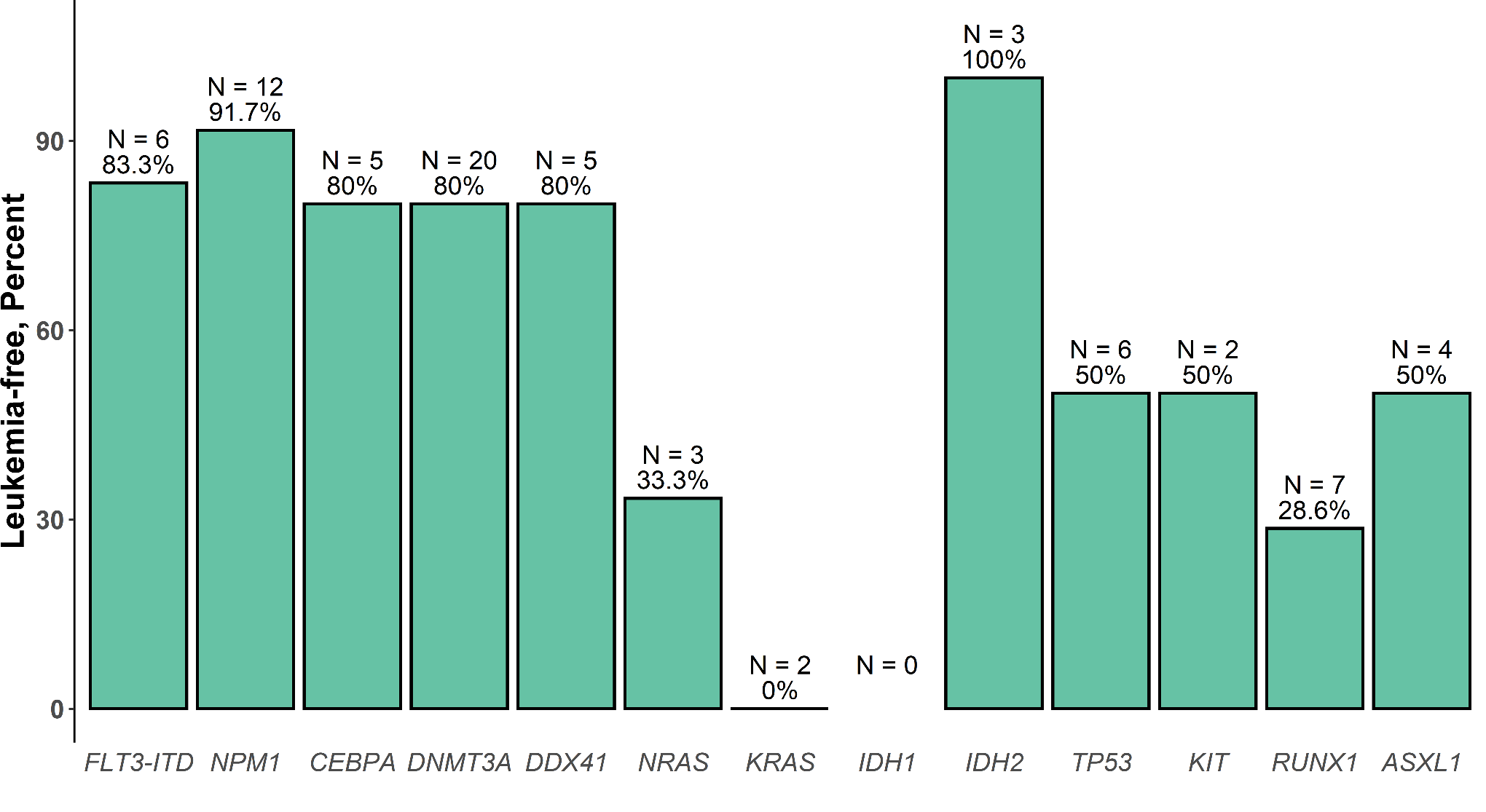
Supplementary Figure 8. Leukemia-free state by DEC+VEN treatment according to gene mutation. DEC: Decitabine; VEN: Venetoclax

# Supplementary Figure 9. Comparison of overall survival by the Wheatley index and TRM risk in entire DEC and DEC+VEN patients. DEC: Decitabine; HR; Hazard ratio; TRM: Treatment-related mortality; VEN: Venetoclax

**
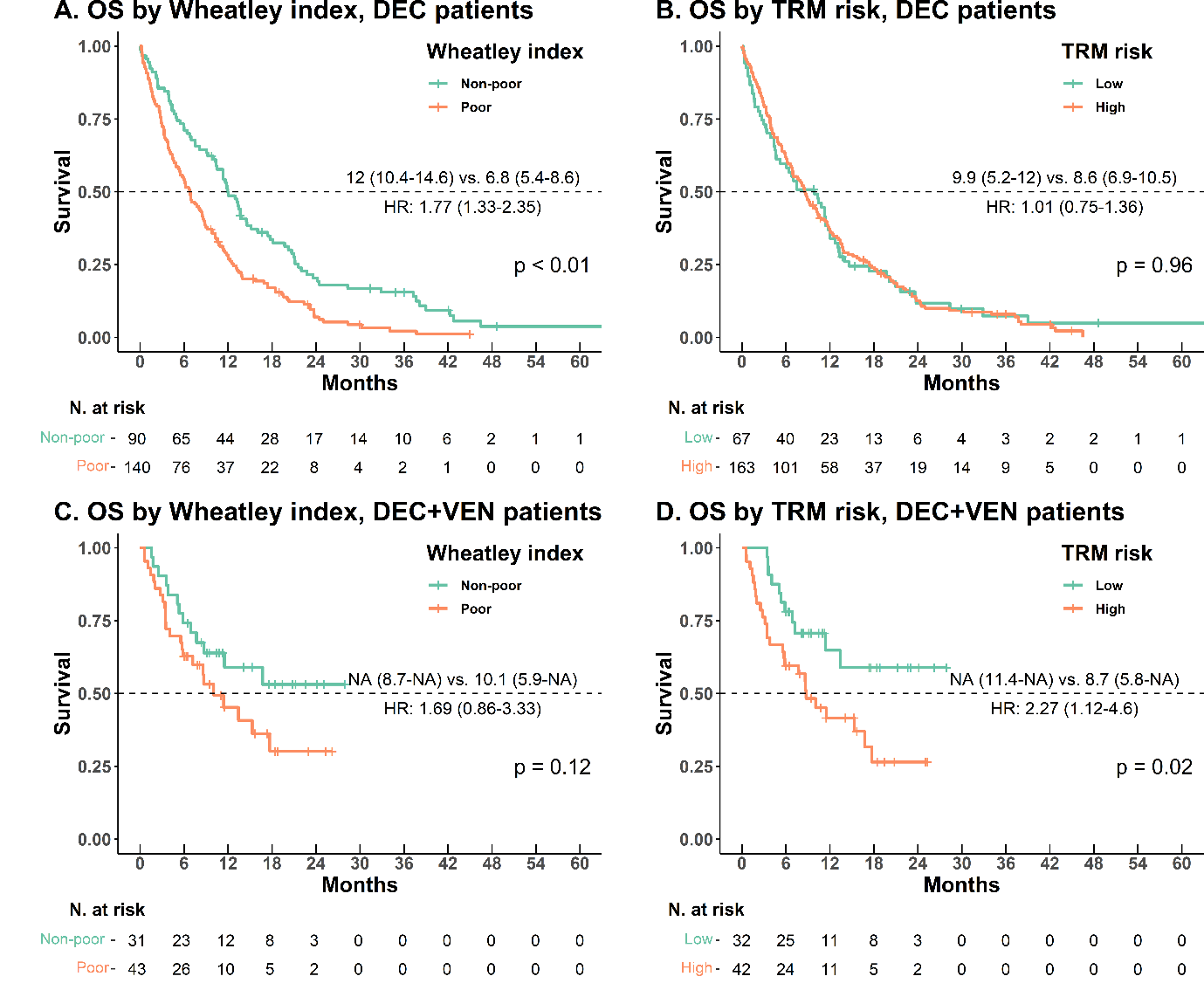
**
